# Enhanced response obtainability with chirp stimulus in bone-conducted mVEMPs

**DOI:** 10.1007/s00405-026-10332-7

**Published:** 2026-06-01

**Authors:** Ceren Karaçaylı, Ercan Karababa, Emine Ceren Ersöz Ünlü, Yavuz Fuat Yılmaz

**Affiliations:** 1 Department of Audiology, University of Health Sciences, Gülhane Facultyof Health Science, Gülhane Eğitim ve Araştırma Hastanesi KBB AD, Ankara, Turkey; 2 Department of Otorhinolaryngology, Gülhane Training and Research Hospital, Ankara, Turkey

**Keywords:** VEMP, Masseter, Bone conducted, Tone burst, Chirp

## Abstract

**Purpose:**

Masseter vestibular-evoked myogenic potentials (mVEMPs) assess vestibular function through the vestibulo-masseteric reflex. While tone burst (TB) stimuli are commonly used, narrowband chirp stimuli may improve response rates and neural synchronization. This study compares the effectiveness of TB and chirp stimuli in bone-conducted mVEMP testing.

**Methods:**

Thirty-five healthy participants (70 ears) underwent mVEMP testing using a bone conduction vibrator. We evaluated response obtainability, P1 and N1 latencies, and P1-N1 amplitude in bone-conducted mVEMPs using 500 Hz TB and 500 Hz narrowband level-specific (NB LS) chirp stimuli.

**Results:**

The response rate for the NB LS chirp stimulus (80.0%) was significantly higher than that for the TB stimulus (42.9%) (*p* < 0.001). Logistic regression analysis indicated that the likelihood of obtaining a response with chirp stimuli was 5.333 times higher than with TB stimuli (*p* < 0.001). Additionally, chirp stimuli produced significantly shorter latencies and higher amplitudes (*p* < 0.001).

**Conclusion:**

In bone-conducted mVEMPs, NB LS chirp stimuli improve response rates, shorten latencies, and increase amplitudes, suggesting greater reliability and efficiency in vestibular assessment.

## Introduction

Masseter vestibular-evoked myogenic potentials (mVEMPs) allow clinicians to probe vestibular function through the vestibulo-masseteric reflex pathway. This reflex is mediated via saccular afferents that project through the inferior vestibular nerve to the trigeminal motor nucleus, ultimately activating the masseter muscle [1–5]. Unlike cervical VEMPs (cVEMPs), which reflect the vestibulo-collic reflex via the sternocleidomastoid muscle, mVEMPs offer a distinct window into brainstem-level vestibular processing. mVEMPs are recorded in response to auditory stimuli — typically loud clicks or tone bursts — and produce a characteristic biphasic waveform that reflects the integrity of this pathway [1, 6].

Beyond their role in vestibular assessment, mVEMPs have demonstrated clinical utility in the evaluation of neurological conditions affecting the brainstem, including Parkinson’s disease, where the symmetry and amplitude of mVEMP responses may help differentiate peripheral from central vestibular pathology [1, 7]. Established normative data and favorable test-retest reliability further support their clinical applicability as a non-invasive tool in the diagnostic workup of vestibular and brainstem disorders [8–11].

The application of 500 Hz tone burst and 500 Hz chirp stimuli in vestibular-evoked myogenic potentials (VEMPs) has attracted considerable interest owing to their efficacy in generating strong responses from the vestibular system. The 500 Hz tone burst is acknowledged as an ideal stimulus for VEMP testing, as it generally yields lower stimulus thresholds and greater amplitudes than click stimuli, rendering it a favored option in clinical environments [12, 13]. In contrast, the 500 Hz NB LS chirp stimulus is intended to more effectively distribute sound energy across a range of frequencies. This makes it a viable alternative to traditional tone bursts. Chirp stimuli, such as the narrowband CE-chirp, are thought to improve the strength and timing of VEMP responses. However, studies employing air-conducted stimuli indicate that while chirp stimuli consistently produce shorter latencies, they do not demonstrate a significant amplitude advantage over the 500 Hz tone burst in cVEMP testing [14, 15]. In particular, for mVEMP via air conduction, Neupane et al. found shorter latencies with NB CE-chirp compared to tone burst without significant difference in amplitude [16], whereas Nagarajan et al. observed shorter latencies and larger amplitudes favoring chirp stimulation [17]. To date, whether the advantages of chirp stimulation extend to bone-conducted mVEMP testing remains unknown, as no such investigation has been reported in the literature.

Bone-conducted stimuli have become an effective technique for VEMPs, especially in clinical situations where air-conducted hearing is impaired, such as in conductive hearing loss or middle ear disorders. Unlike air-conducted stimuli, bone conducted vibration (BCV) bypasses the outer and middle ear entirely, delivering vibrations directly to the vestibular apparatus — an advantage in cases where middle ear pathology may otherwise compromise sound transmission [18, 19]. In individuals with normal hearing, the responses obtained through bone conduction are broadly comparable to those elicited by air-conducted stimuli [20, 21]. This makes BCV particularly useful in clinical populations such as those with otosclerosis, where the vestibular system may not be adequately reached through conventional air-conducted approaches [22, 23].

It is important to emphasize that mVEMPs and cVEMPs assess fundamentally distinct reflex pathways. While cVEMPs reflect the vestibulo-collic reflex mediated via the sternocleidomastoid muscle and the spinal accessory nerve, mVEMPs probe the vestibulo-masseteric reflex, which is mediated through projections to the trigeminal motor nucleus [1–3]. These pathways differ in their neural generators, muscle activation patterns, and central relay stations; therefore, findings obtained from cVEMP testing cannot be directly extrapolated to mVEMP testing.

While chirp stimuli have been evaluated in bone-conducted oVEMP [24] and cVEMP [25] testing with favorable results, no study has yet investigated their use in bone-conducted mVEMP testing. The objective of this study was therefore to compare the response obtainability, P1 latency, N1 latency, and P1-N1 amplitude of a 500 Hz Tone Burst stimulus and a 500 Hz Narrowband Level-Specific (NB LS) chirp stimulus in bone-conducted mVEMP testing.

## Materials and methods

All procedures adhered to the Declaration of Helsinki and received approval from the University’s Ethics Committee prior to the enrollment of the first participant (approval date: December 10, 2024; approval number: 2024/567). All participants provided written informed consent before participation. Data collection was conducted between January and March 2025. Thirty-five healthy participants (seventy ears) were included, comprising twenty-two females and thirteen males. Prior to commencing the test, all participants underwent otological evaluation, audiometric assessment, and tympanometric testing. Participants were excluded if they had a history of vestibular disorders (e.g., Menière’s disease, benign paroxysmal positional vertigo, or vestibular neuritis), neurological conditions (e.g., multiple sclerosis, Parkinson’s disease, or brainstem pathology), or use of centrally acting medications (e.g., antiepileptics or antidepressants). Additional exclusion criteria included temporomandibular joint (TMJ) dysfunction, bruxism, masseter muscle or masticatory system pathology, facial nerve paralysis, history of otological surgery, active ear infection, and systemic diseases such as diabetes mellitus or autoimmune disorders. Only participants with normal hearing thresholds and normal tympanometric findings were included.

The mVEMP responses were obtained using the Eclipse EP 25 (Interacoustics A/S, Middelfart, Denmark). Participants were directed to adopt a seated position guaranteeing ideal comfort during the testing process. The participants’ skin was carefully cleaned with ethyl alcohol before electrodes were applied to guarantee sterility and eliminate any last traces of contaminants. Electrode impedances were maintained below 5 kΩ. Participants in the mVEMP testing procedure were directed to activate the masseter muscle by clenching their posterior teeth. Zygomatic electrode placement was utilized in mVEMP recordings [2]. The active electrode was placed over the lower third of masseter muscle, the reference electrode was positioned on the zygomatic arch, and the ground electrode was located on the forehead. Participants were instructed to maintain this position throughout the test, with periodic verbal feedback provided as needed. Following every recording, a period of rest spanning several minutes was allocated to prevent the development of masseter muscle fatigue. Tone bursts and NB LS chirp stimuli at 500 Hz were presented in a random sequence using a bone conduction vibrator (B71 model; Radioear Company, Minnesota/USA) positioned on the mastoid region.

The 500 Hz tone burst was stimulated at a strength of 50 dB nHL and a rate of 5.1 stimuli per second. The analysis duration was 55 ms. Two hundred stimuli were averaged using a rarefaction polarity. The rise, plateau, and fall durations of the stimulus were 2 ms, 1 ms, and 2 ms, respectively. The high-pass filter was configured to 10 Hz, while the low-pass filter was set to 1000 Hz.

500 Hz NB LS chirp stimulus extended from 360 to 720 Hz, was delivered at an intensity of 50 dB nHL, and maintained for 9 ms. The stimulus rate was established at 5.1/s, the analysis duration at 55 ms, and the polarity was set to rarefaction. Two hundred stimuli were averaged. The position of the bone conduction vibrator was kept constant between the TB and NB LS chirp stimulus recordings.

The stimulus intensity was set at 50 dB nHL for both stimuli, as this represents the maximum output of the bone conduction vibrator (B71, Radioear) used in this study. This intensity limitation should be considered when interpreting response obtainability rates, particularly in comparison with studies employing higher stimulus levels.

### Statistical analysis and sample size calculation

Sample size was estimated based on a priori power analysis with G*Power software and reference values from a previous bone-conducted VEMP study using the same stimuli [24]. A Wilcoxon signed-rank test (A.R.E. method) with an effect size of dz = 0.779 (P1-N1 amplitude, the most conservative estimate), α = 0.05, and a power of 0.95 required a minimum sample size of 21 participants. The final sample of 35 participants was above this requirement to ensure sufficient statistical power to detect differences in all outcome measures.

All statistical analyses were performed using SPSS (IBM SPSS Statistics, Version 25). The McNemar test was applied to assess the statistical significance of differences between the TB and NB LS chirp stimulus in response obtainability, as both methods were applied to the same participant group, and the data were categorical.

A binary logistic regression analysis was performed to evaluate the effect of stimulus type (NB LS chirp vs. TB) on response obtainability (Response vs. No Response). The stimulus type was included as a categorical covariate, with TB stimulation set as the reference category. The dependent variable was response status, categorized as Response (1) or No Response (0).

The logistic regression model was assessed with the Omnibus test of model coefficients, -2 Log Likelihood and Nagelkerke R^2^ to check model fit and explanatory power. The type of stimulus was quantified for its effect on the probability of a response by calculating odds ratios (Exp(B)) with 95% confidence intervals (CIs). The fit of the model was further assessed by the Hosmer-Lemeshow test and a contingency table of observed versus expected values.

Normality of continuous variables was evaluated using the Shapiro-Wilk test. The statistical methods used for comparison were determined based on the distribution of the data. P1 latency and N1 latency were analyzed with Wilcoxon Signed-Rank Test since the data did not fulfill the assumption of normality. The P1-N1 amplitude was normally distributed, therefore it was compared using a paired (repeated samples) t-test.

All statistical tests were two-sided, with statistical significance set at *P* < 0.05.

## Results

This study included 35 participants (70 ears) with a mean age of 31.34 ± 10.101, ranging from 21 to 53 years. Of the participants in the study, 22 were female and 13 were male.

**Fig. 1:**
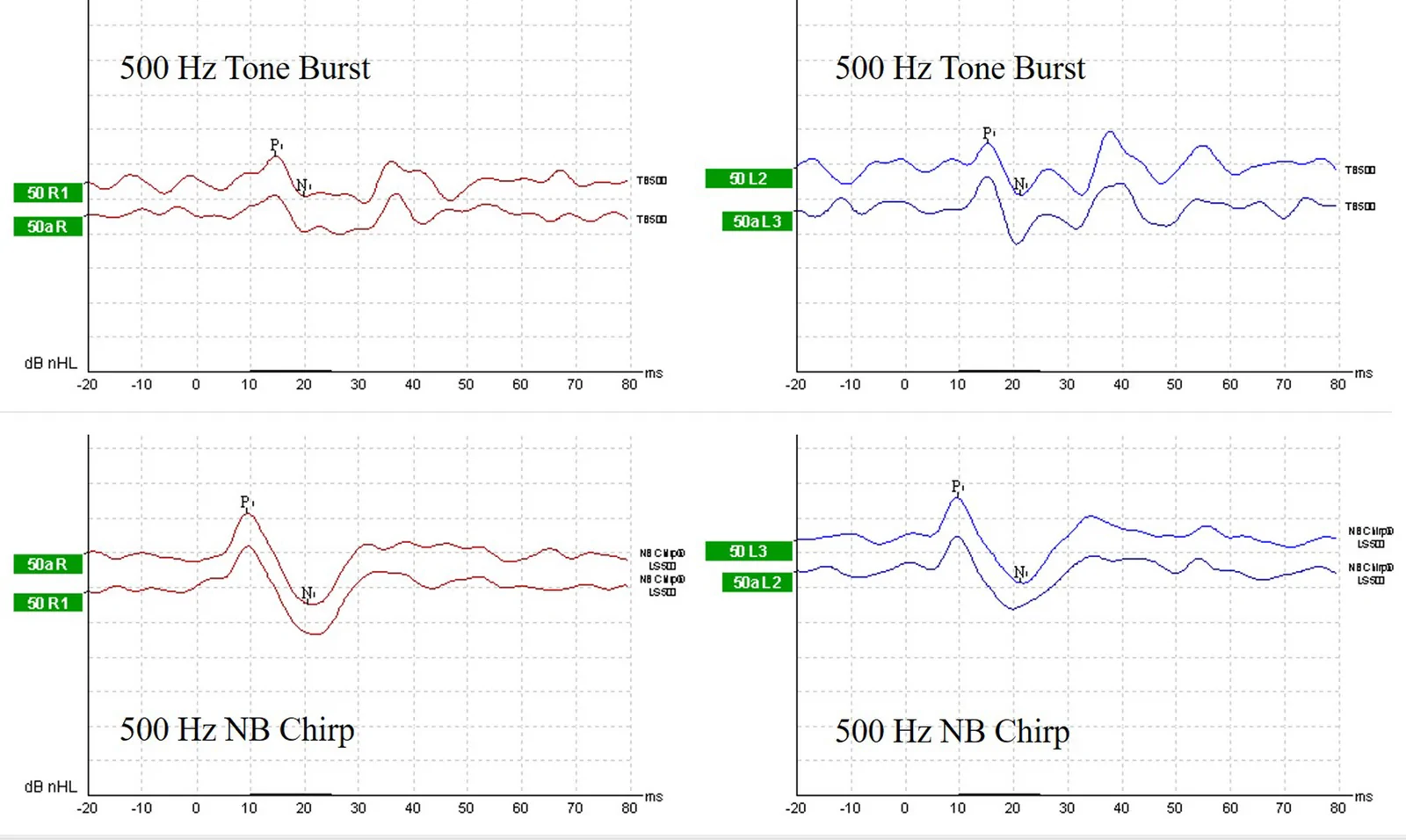
Masseter VEMP Waveforms: 500 Hz Tone Burst vs. 500 Hz NB LS Chirp Stimulus

A significant difference was observed in the proportion of responses between the tone burst and NB LS chirp stimulation. The proportion of cases classified as “response present” was 42.9% (30/70) for the tone burst stimulus and 80.0% (56/70) for the chirp stimulus. Wave samples obtained with TB and NB LS chirp stimulation from the same participant are shown in Fig. 1.

The paired-samples McNemar test revealed a significant difference in response classification between the two stimuli (Z = -4.747, *p* < 0.001). The proportion difference between the tone burst and chirp stimuli was − 0.371 (95% CI: -0.498 to -0.232), indicating that the chirp stimulus resulted in a significantly higher response rate compared to the tone burst stimulus.

Notably, 28 participants (40.0%) were categorized as “response present” by the chirp stimulus but “response absent” by the tone burst stimulus, while only 2 participants (2.9%) were identified as “response present” by the tone burst stimulus but “response absent” by the chirp stimulus. Comparison of Response Rates Between 500 Hz Tone Burst and NB LS chirp Stimulus is shown in Table 1.

**Table 1:** Comparison of Response Rates Between 500 Hz Tone Burst and NB LS Chirp Stimuli

		500 Hz NB LS Chirp			p
		No Response (-)	Response (+)	Total	
500 Hz Tone Burst	No response (-)	12 (30%)	28 (70%)	40 (57.1%)	< 0.001
	Response (+)	2 (6.7%)	28 (93.3%)	30 (42.9%)	
	Total	14 (20%)	56 (80%)	70 (100%)	

*McNemar Test

A binary logistic regression analysis was conducted to assess the effect of stimulus type (NB LS chirp vs. TB) on response probability (Response vs. No Response). The omnibus test of model coefficients confirmed that the model was statistically significant (χ² = 21.038, df = 1, *p* < 0.001), indicating that stimulus type significantly influenced response probability.

Model fit assessment showed a -2 Log Likelihood of 165.663, with Cox & Snell R² = 0.140 and Nagelkerke R² = 0.189, suggesting that the model explains approximately 18.9% of the variance in response probability. Although the Hosmer-Lemeshow test could not be computed (df = 0, p = .), the contingency table showed a close match between observed and expected values, supporting an acceptable model fit.

Odds ratio analysis demonstrated that chirp stimulation significantly increased the likelihood of response compared to TB stimulation. Specifically, the odds of obtaining a response with chirp stimulation were 5.333 times higher than with TB stimulation (OR = 5.333, 95% CI: 2.512–11.325, *p* < 0.001). This suggests that participants undergoing chirp stimulation were more than five times as likely to respond compared to those undergoing TB stimulation.

Latency and amplitude comparisons were restricted to ears that yielded responses to both stimuli (*n* = 28 ears). When P1 latency, N1 latency, and P1-N1 amplitude were compared between 500 Hz Tone Burst and 500 Hz NB LS chirp stimulation, the P1 latency was significantly shorter with NB LS chirp (12.99 ± 3.3 ms, median: 11.17 ms [range: 9–19]) compared to Tone Burst (18.34 ± 3.52 ms, median: 16.84 ms [range: 13.67–24.67]) (Wilcoxon Signed Rank Test, Z = -4.628, *p* < 0.001). Similarly, the N1 latency was also significantly shorter with NB LS chirp (18.96 ± 4.3 ms, median: 19.67 ms [range: 13–25.67]) compared to Tone Burst (23.36 ± 3.66 ms, median: 24.17 ms [range: 17.33–29]) (Wilcoxon Signed Rank Test, Z = -4.278, *p* < 0.001).

In contrast, the P1-N1 amplitude was significantly higher with NB LS chirp (63 ± 20.94 µV, median: 63.11 µV [range: 22.34–102.1]) compared to Tone Burst (42.54 ± 14.87 µV, median: 40.66 µV [range: 19.28–84.46]) (Paired Samples T-Test, t = -5.142, *p* < 0.001).

These findings indicate that NB LS chirp stimulus elicits significantly shorter latencies and greater amplitudes compared to the Tone Burst stimulus, suggesting enhanced neural response characteristics. Comparison of P1 Latency, N1 Latency, and Amplitude Between 500 Hz Tone Burst and 500 Hz NB LS chirp stimuli was shown in Table 2.

**Table 2:** Comparison of P1 Latency, N1 Latency, and Amplitude Between 500 Hz Tone Burst and 500 Hz NB LS Chirp Stimuli

	500 Hz Tone burst		500 Hz NB LS Chirp		Test Stats.	*p*
	Mean ± SD	Median (min-max)	Mean ± SD	Median (min-max)		
p1 Latency (ms)	18.34 ± 3.52	16.84 (13.67–24.67)	12.99 ± 3.3	11.17 (9–19)	-4.628	< 0.001*
n1 Latency (ms)	23.36 ± 3.66	24.17 (17.33-29)	18.96 ± 4.3	19.67 (13-25.67)	-4.278	< 0.001*
Amplitude (µV)	42.54 ± 14.87	40.66 (19.28–84.46)	63 ± 20.94	63.11 (22.34–102.1)	-5.142	< 0.001**

*Wilcoxon Signed Rank Test, **Paired Samples *T* Test, *ms* millisecond, *µV* microvolt, *SD* standard deviation, *min* minimum, *max* maximum

## Discussion

Bone-conducted stimuli have demonstrated efficacy in activating the vestibular system, circumventing potential obstructions posed by the outer and middle ear. This is particularly advantageous for participants with conductive hearing loss because air-conducted stimuli may not sufficiently stimulate the vestibular apparatus [20]. Studies have shown that bone-conducted VEMPs can produce robust responses often at lower sound levels than air-conducted stimuli, making the vestibular assessment more reliable in difficult clinical situations. For example, Shabana et al. demonstrated that bone-conducted VEMP responses were preserved in subjects with chronic suppurative otitis media and the usefulness of the method in populations where classical air-conducted methods may fail [20].

This study examined which stimulus is most effective for eliciting mVEMP responses via bone conduction, revealing that the 500 Hz NB LS stimulus yielded a higher response rate. To our knowledge, only one study exists in the literature that compares mVEMP responses utilizing bone conduction stimuli. This study by Kumar et al. compared a 500 Hz tone burst stimulus and a click stimulus, revealing a 100% response rate for both stimuli in neurotypical adults [21]. The stimulus intensity in this study was 65 dB nHL. The stimulus level in our study was set at 50 dB nHL. The disparity in obtainability rates between the two studies may be attributable to this distinction.

The integration of chirp stimuli, especially narrowband chirps, has been investigated in relation to VEMP testing. Chirp stimuli are engineered to transmit sound energy more effectively across a frequency spectrum, potentially increasing the amplitude and decreasing the latency of VEMP responses. The application of chirp stimuli alongside bone conduction may enhance the elicitation of VEMPs, offering a more thorough assessment of vestibular function. Narrow band chirps have been demonstrated in recent studies to elicit larger amplitudes of VEMP responses than traditional tone bursts and thus may be a possible tool for improved diagnostic accuracy [26]. In a study by Neupane et al., for air-conducted mVEMP studies, the 500 Hz NB CE-chirp was found to have significantly shorter latencies than tone burst stimuli, while there was no significant difference in amplitude [16]. On the other hand, recordings of mVEMP in air conduction reported shorter latencies and larger amplitudes for chirp stimulation compared to tone bursts [17]. Interestingly, a meta-analysis of studies of air-conducted cVEMP confirmed that although chirp consistently produces shorter latencies, it does not provide a significant amplitude advantage compared to tone burst stimulation [15]. The present study extends these findings to the bone-conducted modality, where chirp stimulation yielded not only shorter latencies but also significantly larger P1-N1 amplitudes. This discrepancy may reflect the unique mechanical coupling properties of bone conduction, which bypasses middle ear transmission and allows more direct stimulation of the vestibular apparatus, potentially enhancing the frequency-specific energy delivery of the chirp stimulus.

Çoban et al. employed a bone-conducted 500 Hz tone burst and chirp stimulus in the oVEMP assessment. This study found a 100% response rate for both stimuli; however, p1 and n1 latencies were significantly reduced, and p1n1 amplitude had significantly risen with the chirp stimulus [24]. In a cVEMP study performed at our clinic utilizing bone-conducted 500 Hz tone bursts and chirp stimuli, the response rate for tone bursts was 51.4%, whereas the response rate for chirp stimuli was 95.7%. In this study, p1 and n1 latencies were significantly reduced, and p1n1 amplitude was markedly increased with the chirp stimulus [25]. Notably, although the present study shares the same stimulation modality and general methodological framework with our previous cVEMP investigation [25], the two studies address physiologically distinct reflex arcs — the vestibulo-collic reflex in the case of cVEMPs versus the vestibulo-masseteric reflex in the case of mVEMPs [2, 3] — and therefore their findings should not be regarded as interchangeable or as a unified demonstration of chirp superiority across all bone-conducted VEMPs. The results of these studies are also consistent with the findings of our current study. These findings can be explained by several stimulus-related factors. The tone burst stimulus exhibits a significantly longer rise and fall time, which diminishes its effectiveness. Chirp stimuli differ from tone bursts in that they lack rise and fall times. The latency of reactions in BC oVEMP consists of three components: bone conduction through the skull, transit time within the utricle, and the neural response. Çoban et al. indicated that the chirp stimulus exhibits greater stability compared to the TB stimulus. The long latencies and low amplitudes observed with TB stimuli may be attributed to frequency scattering [24]. The superior performance of chirp stimulation observed in the present study may similarly reflect these stimulus characteristics.

A potential concern with bone-conducted mVEMP recordings is whether the responses reflect vestibular activation or the segmental jaw jerk reflex, which also involves the masseter muscle via the trigeminal motor nucleus. However, several lines of evidence support the vestibular origin of the responses recorded here. First, the P1 latency of the jaw jerk reflex (mean ~ 8.7 ms in healthy individuals) [27] is considerably shorter than the P1 latencies observed in our study for both TB (mean 18.34 ms) and NB LS chirp (mean 12.99 ms) stimuli, making contamination by segmental trigeminal reflexes unlikely. Second, Deriu et al. established that sound-evoked masseteric responses are absent in patients with vestibular lesions but preserved in those with cochlear lesions, confirming the vestibular rather than cochlear or segmental origin of these potentials [3]. Third, bone-conducted vibration applied to the mastoid is not expected to activate periodontal mechanoreceptors or jaw proprioceptors at the stimulus parameters used in this study.

From a clinical perspective, the improved response obtainability achieved with NB LS chirp stimulation in bone-conducted mVEMP testing may have particular relevance in populations where reliable vestibular assessment is challenging. Given that mVEMPs probe the vestibulo-masseteric reflex arc — a pathway involving the trigeminal motor nucleus and brainstem relay stations — their application may be especially valuable in the evaluation of brainstem pathologies, including Parkinson’s disease and multiple sclerosis, where this reflex arc may be selectively affected [1, 6, 7]. The use of chirp stimuli, by increasing response obtainability and signal quality, may therefore enhance the diagnostic sensitivity of mVEMP testing in these neurological conditions.

The primary limitation of this study is that BC stimulation activated both the saccule and the utricle, thereby eliciting a comprehensive response from the entire otolith organ. A further limitation is that the study exclusively involved healthy volunteers. While it is asserted that chirp stimulus is more effective in bone conduction, additional research is required regarding its application in participants with conductive hearing loss. Additionally, the stimulus intensity was limited to 50 dB nHL due to the maximum output of the bone conduction vibrator used, which may have contributed to the lower response rates observed with the tone burst stimulus compared to studies employing higher intensity levels.

## Conclusion

In the mVEMP test utilizing bone-conducted stimuli, the 500 Hz NB LS chirp stimulus yields a higher response rate, shorter P1 and N1 latencies, and larger P1-N1 amplitude compared to the 500 Hz TB stimulus. These findings suggest that the 500 Hz NB LS chirp stimulus is a more reliable and efficient stimulus for bone-conducted mVEMP testing, with potential clinical advantages particularly in populations where air-conducted stimulation is insufficient, such as those with conductive hearing loss or middle ear pathology.
